# Targeting oncogenic miR-335 inhibits growth and invasion of malignant astrocytoma cells

**DOI:** 10.1186/1476-4598-10-59

**Published:** 2011-05-19

**Authors:** Minfeng Shu, Xiaoke Zheng, Sihan Wu, Huimin Lu, Tiandong Leng, Wenbo Zhu, Yuehan Zhou, Yanqiu Ou, Xi Lin, Yuan Lin, Dong Xu, Yuxi Zhou, Guangmei Yan

**Affiliations:** 1 Department of Pharmacology, Zhongshan School of Medicine, Sun Yat-Sen University, Guangzhou, China

## Abstract

**Background:**

Astrocytomas are the most common and aggressive brain tumors characterized by their highly invasive growth. Gain of chromosome 7 with a hot spot at 7q32 appears to be the most prominent aberration in astrocytoma. Previously reports have shown that microRNA-335 (miR-335) resided on chromosome 7q32 is deregulated in many cancers; however, the biological function of miR-335 in astrocytoma has yet to be elucidated.

**Results:**

We report that miR-335 acts as a tumor promoter in conferring tumorigenic features such as growth and invasion on malignant astrocytoma. The miR-335 level is highly elevated in C6 astrocytoma cells and human malignant astrocytomas. Ectopic expression of miR-335 in C6 cells dramatically enhances cell viability, colony-forming ability and invasiveness. Conversely, delivery of antagonist specific for miR-335 (antagomir-335) to C6 cells results in growth arrest, cell apoptosis, invasion repression and marked regression of astrocytoma xenografts. Further investigation reveals that miR-335 targets disheveled-associated activator of morphogenesis 1(Daam1) at posttranscriptional level. Moreover, silencing of endogenous Daam1 (siDaam1) could mimic the oncogenic effects of miR-335 and reverse the growth arrest, proapoptotic and invasion repression effects induced by antagomir-335. Notably, the oncogenic effects of miR-335 and siDAAM1 together with anti-tumor effects of antagomir-335 are also confirmed in human astrocytoma U87-MG cells.

**Conclusion:**

These findings suggest an oncogenic role of miR-335 and shed new lights on the therapy of malignant astrocytomas by targeting miR-335.

## Introduction

Astrocytomas derived from astrocytes or astroglial precursors are the most common malignant cancer affecting the central nervous system, accounting for >60% of primary brain tumors [1]. Current therapies for astrocytomas including surgery, radiation, and chemotherapy have not been successful due to the rapid and invasive tumor growth, the genetic heterogeneity and our poor understanding of the molecular mechanisms governing disease manifestation and progression[2].

MicroRNAs (miRNAs) are small non-coding RNAs (18 to 25 nucleotides) with potential roles in regulation of gene expression at posttranscriptional level[3]. Cumulative evidence suggests that deregulation of miRNAs may contribute to specific human diseases, including cancer. It has been reported the amplification or overexpression of implicated microRNAs in cancers could materially serve as oncogenes[4]. Meanwhile, the tumor suppressing roles of certain miRNAs have also been presumed due to their physical deletion or reduced expression in human cancer[5]. Of note, recent data suggest an advantage of miRNA-based classification than mRNA profiling in origin identifying[6], novel biomarkers for diagnosis[7,8] and prognosis predicting for cancer patients[9]. Even more, miRNAs stand for potential promising therapeutic targets for cancer treatment[7,8,10,11]. These findings provide new insights into the mechanisms of the tumor biology and give a novel thought to the therapeutic strategies

It is well established that chromosome 7q32 is a hot spot that frequently amplified in malignant astrocytomas[12]. There are 8 miRNAs(miR-593, miR-129-1, miR-335, miR-182, miR-96, miR-183, miR-29a, miR-29b-1) resided on this genomic locus, some of which have been investigated, either as oncogenes or tumor suppressor genes [13-15]. MiR-335, which is transcribed from the genomic region chromosome 7q32.2, has been reported to act as a tumor initiation and metastasis suppressor of breast cancer[16,17]. Furthermore, it is also demonstrated that miR-335 regulates Rb1 and controls cell proliferation in a p53-dependent manner[18]. In addition, a recent study has shown that miR-335 orchestrates cell proliferation, migration and differentiation in human mesenchymal stem cells[19]. These investigations indicate the important roles of miR-335 in tumor initiation and progression; however, the biological role of miR-335 in malignant astrocytoma pathogenesis is still largely unknown.

In this study, we aimed to investigate the possible contributions of miR-335 imbalance to astrocytoma pathogenesis. We found that miR-335 targeted a potential tumor suppressor Daam1 in astrocytoma cells, which promoted several malignant features such as growth and invasion, whereas miR-335 inhibition could potently induce growth arrest, apoptosis and invasion repression both *in vitro *and *in vivo*. These findings suggest an oncogenic role of miR-335 in the molecular etiology of malignant astrocytomas and might provide new insights into the implication for future cancer therapy.

## Materials and methods

### Cell Cultures and Patient Tissues

Rat C6 astrocytoma cells and human U87-MG astrocytoma cells were obtained from the American Type Culture Collection. Human HEB normal astrocytes were obtained from Guangzhou Institutes of Biomedicine and Health, Chinese Academy of Sciences (Guangzhou, China). Cell cultures were performed as described previously[20]. Briefly, cells maintained in DMEM (Invitrogen, USA) supplemented with 10% FBS and a humidified atmosphere of 5% CO_2 _at 37°C. Rat normal astrocytes were obtained from Sprague-Dawley rat pups (postnatal 7~8 days). The first three passage astrocytes were used in our study. Tissue specimens (tumor, adjacent paracancerous tissues) of malignant astrocytoma patients were collected after informed consent and immediately frozen in liquid nitrogen. The astrocytoma tissues were verified by a pathologist as WHO grade II-III and the patients' characteristics were indicated in Additional file 1 Table S1.

### Cell Viability Assay

Cell viability was determined by the 3-(4,5-dimethylthiazol-2-yl)-2,5-diphenyl tetrazolium bromide (MTT, Sigma, USA) assay. Briefly, cells were seeded in 96-well plates at 2 × 10^3 ^cells/well and incubated overnight. MTT solution (5 mg/ml in PBS, 20 μl/well) was added to the cells to produce formazan crystals. MTT solution was substituted by 150 μl DMSO 4 h later to solubilize the formazan crystal. The optical absorbance was determined at 490 nm using an iMark microplate reader (Bio-Rad, USA).

### Oligonucleotide Transfection

MiR-335 mimics, negative control (NC), antagomir-335, antagomir-NC and Daam1 silencing oligonucleotides (siDaam1) (RiboBio, China) were transfected using Lipofectamine RNAiMAX (Invitrogen) according to manufacturer suggested procedures. Transfection efficiency was evaluated by Cy3-labeled oligonucleotides negative control (data not shown).

### Colony Formation Assay

Colony formation was evaluated as previously described [21]. Twenty-four hours after transfection, 200~500 transfected cells were placed in a fresh six-well plate and maintained in DMEM containing 10% FBS for 7 to 10 days. Colonies were fixed with methanol and stained with 0.1% crystal violet in 20% methanol for 15 min, and representative colonies were photographed.

### Cell Invasion Assay

Cell culture chamber with 8 μm pore size polycarbonate membrane filters (BD Biosciences, USA) were used for cell invasion assay. The filters were pre-coated with 50 μl Matrigel (1.25 mg/ml). Cells transfected with respective RNAs were harvested and seeded 1 × 10^5 ^cells/well with 1% FBS medium in upper chambers which were soaked in the bottom chambers filled with 0.6 ml complete medium. At the same time, equal cells of the each group were plated to 96-well plates for cell number assay (MTT). The chamber was incubated at 37°C for 36 h and then the Matrigel was removed. The invaded cells were fixed with 4% paraform and stained with hematoxylin before photography and calculation. The invasiveness of cells was evaluated by the percentage-of-invasion (invaded cell number/total cell number × 100%).

### Cell-Cycle Analysis

Cells were collected by trypsinization, washed in PBS, and fixed in 70% ethanol for 30 min at 4°C. After washing with PBS, cells were incubated with the DNA-binding dye propidium iodide (50 μg/ml) and RNase (1.0 mg/ml) for 30 min at 37°C in the dark. Finally, cells were washed and red fluorescence was analyzed by a FACSCalibur flow cytometer (BD, Germany) using a peak fluorescence gate to discriminate aggregates.

### Caspase 3/7 Activation Assay, Hoechst Staining, and TUNEL Assay

For caspase 3/7 activation assay, cells were seeded into 96-well plates at 2 × 10^5 ^cells per well and transfected with antagomir-335 or antagomir-NC at the indicated concentration for 72 h. Meanwhile, cells with the same treatment of each group were plated to 96-well plates for cell number assay (MTT). Caspase activity was determined using Caspase-Glo 3/7 Assay (Promega, USA) according to the manufacturer's instructions, and evaluated as follows: caspase activity/cell number × 100%.

For Hoechst 33258 staining, cells were fixed with 4% paraform for 10 min, stained with Hoechst 33258 (5 ug/ml, Sigma) for 15 min in the dark, and observed by fluorescence microscopy (Olympus, USA) with a 340 nm excitation filter.

For TUNEL assay, apoptotic cells in 4 μm sections of paraffin-embedded tumor samples or antagomir-335 treated cells were detected by In Situ Cell Death Detection Kit-TMR red (Roche, Germany) according to the manufacturers' instructions and the samples were analysed by fluorescence microscopy (Olympus) with a 540 nm excitation filter. The total number of apoptotic cells was quantified in 5 randomly selected microscopic fields.

### Western Blot Analysis

Western blot was perform as described [22].The following antibodies were used: antibodies against DAAM1(1:500; Santa Cruz Biotechnology, USA), GFAP, phosphorylation(Ser 19) of MLC (1:1,000; Cell Signaling Technology, USA) and β-actin (1:2,000; New England Biolabs, USA).

### Luciferase Assay

The potential binding sites of miR-335 within Daam1 3'-UTR were obtained by TargetScan and PicTar. Synthetic oligos including predicted binding sites were annealed then cloned into Xho I/Not I site of psiCHECK-2 (Promega). C6 cells were transiently transfected with wide type (WT) or mutant (MUT) reporter vector and microRNA using Lipofectmine 2000 (Invitrogen) at the indicated concentrations. Luciferase activity was measured 48 h posttransfection using the Dual-Luciferase Reporter Assay System (Promega) followed the manual. Renilla luciferase activity was normalized to corresponding firefly luciferase activity and plotted as a percentage of the control.

### Real-Time PCR-Based Detection of MiR-335 and Rat Daam1-mRNA

Total RNA was prepared using TRIZOL reagent (Invitrogen). Expression of mature miR-335 was determined by stem-loop primer SYBR Green quantitive real time-PCR (qRT-PCR) and normalized to U6. The stem-loop primer sequence for reverse transcription was as follows: 5'-CTC AAC TGG TGT CGT GGA GTC GGC AAT TCA GTT GAG ACA TTT TT-3'. Rat Daam1 mRNA expression was detected by qRT-PCR and normalized to 18SrRNA. The generated cDNA was amplified with primers for miR-335 (5'-ACA CTC CAG CTG GGT CAA GAG CAA TAA CGA AA-3' and 5'-CTC AAC TGG TGT CGT GGA-3') for rat Daam1 (5'-ACT ACT ACA CCA GCA CCA-3' and 5'-ACT CTC CTC ACT TCC ATG-3'). All qRT-PCR were performed in triplicates.

### In Vivo Assay

For antagomir pretreated study, antagomir-335-and antagomir-NC-transfected C6 cells (1 × 10^6 ^) were prepared in 100 μl serum free DMEM medium and then s.c. injected into either side of the posterior flank of each female BALB/c athymic 4-week nude mouse. Tumor growth was monitored by caliper measurement every two days for 2 weeks. Tumor volume (V) was calculated as follows: V = L × W^2 ^× 0.5; L, length; W, width. Tumor weight was detected at the end of the study.

For antagomir treatment, 4-week old athymic nude mice were s.c. injected with 1 × 10^5 ^C6 cells; after approximately 10 days, when tumors became palpable, the tumor-bearing nude mice were treated with antagomir-335. 50 μl of antagomir-335 (diluted in PBS at 4 mg/ml), or control antagomir-NC were injected intratumorally every two days for 2 weeks. Tumor growth was monitored every two days for 3 weeks. Tumor volume (V) and weight was calculated as described above.

### Confocal microscopy

Cells were grown on glass coverslips to 30% confluence and transfected with RNAs for 36 h. The mediums were removed and cells were rinsed with PBS twice. Cells were fixed with 4% paraformaldehyde for 30 min, washed three times with PBS and then penetrated and blocked with PBS containing 0.2% Triton X-100 and 5% bovine serum albumin for 1 h. The blocking buffer was removed and incubated with phallotoxins(5 Units/ml, Molecular probe, USA,) over night at 4°C. Cells were washed three times with penetrating buffer. Images were photographed with a Zeiss LSM 510 Confocal Microscope (Carl Zeiss Microimaging, Thornwood, NY).

### Immunohistochemistry

Immunohistochemistry staining of 4 μm sections of paraffin-embedded samples were performed as described [23]. Sections were stained with primary antibody DAAM1 (1:50) overnight at 4°C, bio-anti mouse IgG for 1 h and then incubated with avidin biotin-peroxidase complex diluted in NaCl ⁄ Pi.

### Statistical Analysis

Data are presented as mean ± SD of three separated experiments if not noticed. A difference with a *P *value < 0.05 by ANOVA was considered statistically significant.

## Results

### MiR-335 is highly expressed in malignant astrocytoma

**Figure 1:**
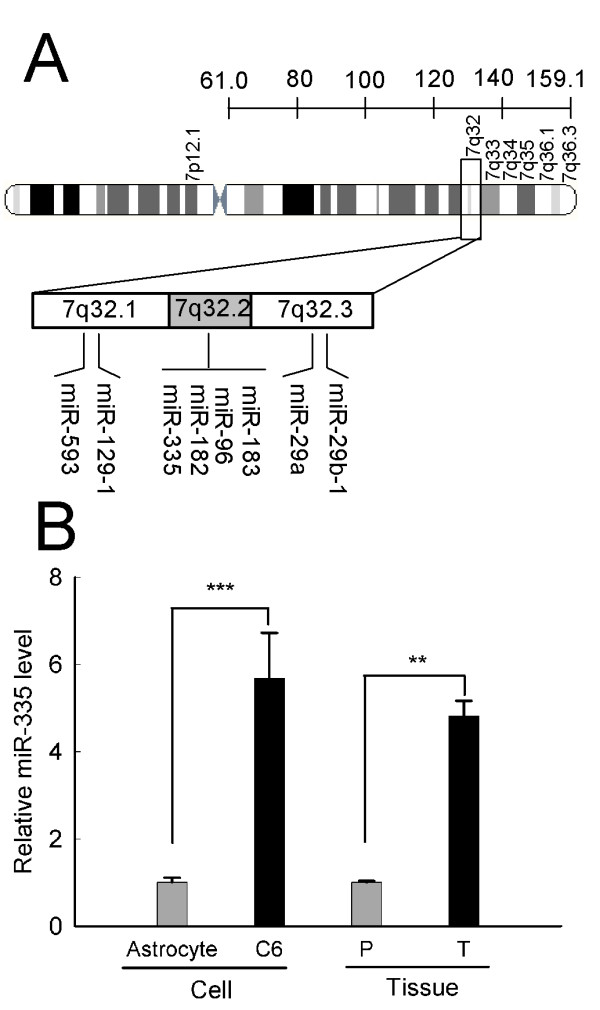
**MiR-335 is highly expressed in astrocytoma**. (A) A schematic showing miRNAs located on chromosome 7q32 in astrocytoma. (B) Data shown are expression level of miR-335 in C6 astrocytoma cells and patient astrocytoma tissues by real-time quantitative RT-PCR, comparing with their relative normal counterparts. P, adjacent paracancerous tissues; T, patient astrocytoma tissues. RNA input was normalized by U6 snRNA. Data represent the means ± SD of three independent experiments. Statistical differences compared with the controls are given as **, P < 0.01; ***, P < 0.001.

We found 8 expressed miRNAs (miR-593, miR-129-1, miR-335, miR-182, miR-96, miR-183, miR-29a, miR-29b-1) resided on chromosome 7q32, a hot spot that frequently gained in astrocytoma(Figure 1A). Among these miRNAs, miR-335 expression between C6 astrocytoma cells and rat normal astrocytes was analyzed by qRT-PCR. Interestingly, the expression of miR-335 was markedly upregulated in C6 cells (Figure 1B). To further confirm and extend this finding, we investigated the expression of miR-335 in a subset of human astrocytoma tissues. Consistently, we also observed a significant higher level of miR-335 in astrocytoma patient samples with respect to their paracancerous counterparts (Figure 1B). These results raise a possibility that miR-335 might be an oncomiRNA in malignant astrocytoma and might well have a role in malignant astrocytoma pathogenesis.

### MiR-335 positively regulates viability and invasion of C6 cells *in vitro *

**Figure 2:**
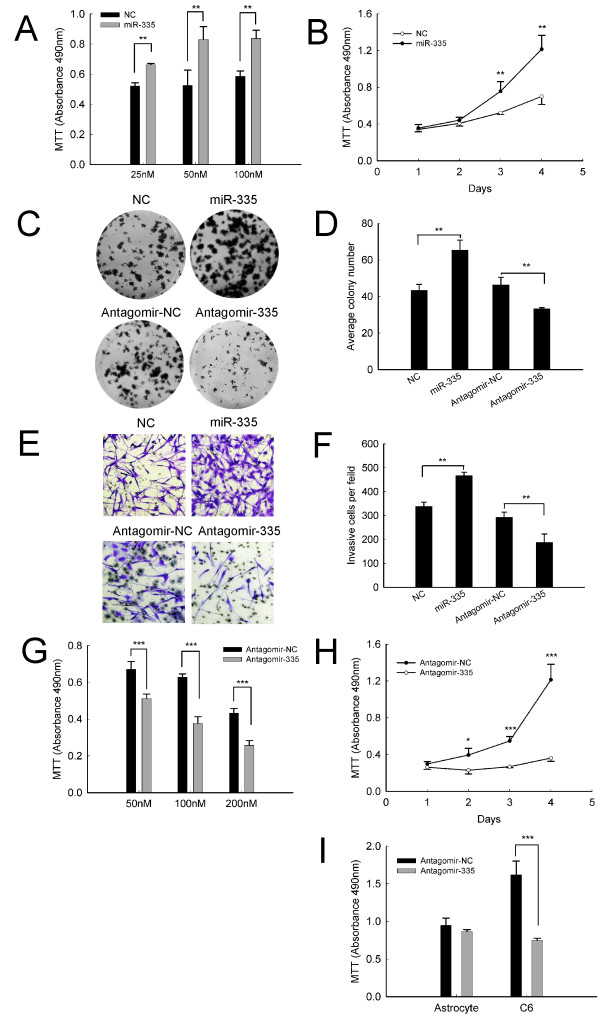
**Effects of miR-335 on viability and invasion in C6 astrocytoma cells**. (A) Dose-dependent effect of miR-335 mimics on cell viability. C6 cells were transfected with miR-335 mimics or negative control mimics (NC) for 72 h at the indicated concentrations. (B) Time-dependent effect of miR-335 mimics on cell viability. C6 cells were transfected with 50 nM miR-335 mimics for the indicated times. (C, D) Colony formation was observed at the presence of miR-335 mimics (C up-panel) or antagomir-335(C down-panel). (E, F) Cell invasiveness was detected by transwell invasion assay. C6 cells were transfected with 50 nM miR-335 mimics (E up-panel) or 100 nM antagomir-335 (E down-panel) or their counterpart negative controls. Graph is the representative of three independent experiments. (G) Dose-dependent effect of antagomir-335 on cell viability. C6 cells were transfected with antagomir-335 or antagomir-NC for 72 h at the indicated concentrations. (H) Time-dependent effect of antagomir-335 on cell viability. C6 cells were transfected with 100 nM antagomir-335 for the indicated times. (I) Cell viability was measured with MTT assay. Rat normal astrocytes and C6 astrocytoma cells were transfected with 100 nM antagomir-335 or antagomir-NC for 72 h. Data represent the means ± SD. Statistical differences compared with the controls are given as *, P < 0.05; **, P < 0.01; ***, P < 0.001. Original magnification in (E), 200 ×.

To elucidate the potential role of miR-335 in astrocytoma pathogenesis, we first performed *in vitro *gain-of-function analyses by introducing miR-335 mimics into C6 cells. Ectogenic miR-335 dramatically enhanced cell viability in dose-and time-dependent manners (Figure 2A, B) and significantly promoted colony formation (Figure 2C up-panel, 2D). Moreover, miR-335 overexpression could also result in marked increase of cell invasiveness (Figure 2E up-panel, 2F). Notably, miR-335 overexpression failed to transform but strongly enhanced the oncogenic properties such as the invasiveness and viability of primary cultured normal astrocytes (Additional file 2 Figure S1).

To determine the consequence of miR-335 inhibition in astrocytoma cells, we delivered an antagonist specific for miR-335 (antagomir-335) into C6 cells. Effective miR-335 inhibition strongly suppressed cell growth (Figure 2G, H), colony formation (Figure 2C down-panel, 2D) and cell invasion (Figure 2E down-panel, 2F). Importantly, knockdown of miR-335 exhibited little sign of viability inhibition on normal astrocytes (Figure 2I), suggesting a specific anti-tumor effect of miR-335 inhibition. Heretofore, all the results authentically indicate an oncomiRNA character of miR-335 as promoting oncogenic phenotypes in astrocytoma.

### MiR-335 abrogation induces apoptosis in C6 cells

**Figure 3:**
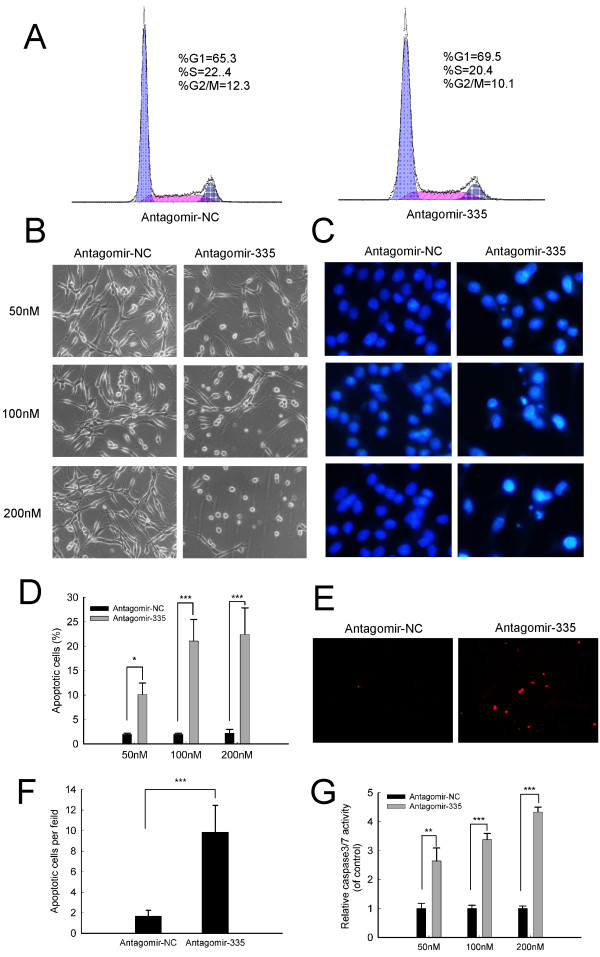
**MiR-335 abrogation induces apoptosis in C6 astrocytoma cells**. (A) Cell cycle distribution detection. C6 cells were transfected with 100 nM antagomir-335 or antagomir-NC for 72 h and cell cycle distribution was analyzed by flow cytometer. (B) Phase-contrast imaging and (C) Hoechest 33258 staining of C6 cells. (D) Quantitation of the percentage of apoptotic cells. (E, F) Apoptotic cells were detected by TUNEL assay. (G) Dose-dependent effect of antagomir-335 on caspase3/7 activity. C6 cells were transfected with antagomir-335 at the indicated concentrations for 72 h. Data represent the means ± SD. Statistical differences compared with the controls are given as *, P < 0.05; **, P < 0.01; ***, P < 0.001. Original magnification in (B, E), 200 ×; Original magnification in (C), 640 ×.

The growth-inhibitory effect of antagomir-335 led us to further investigate whether miR-335 silencing may inhibit cell proliferation and/or induce apoptosis. By flow cytometry analysis, we observed no change on cell cycle distribution (Figure 3A). Nevertheless, apoptotic cells with characteristic morphologic alterations such as cell shrinkage were observed (Figure 3B). Specifically, Hoechst staining and quantification of the number of cells with condensed and fragmented nuclei indicated that antagomir-335 treated cells underwent apoptosis (Figure 3C, D). In line with this observation, TUNEL assay also showed an increased apoptosis in antagomir-335 treated C6 cells (Figure 3E, F).Furthermore, caspase 3/7 activity was markedly enhanced in a dose-dependent manner after 72 h antagomir-335 tranfection, confirming miR-335 abrogation-induced apoptosis (Figure 3G). These results suggest that antagomir-335 induced growth arrest was, at least in part, caused by apoptosis.

### Daam1 is a direct target of miR-335

**Figure 4:**
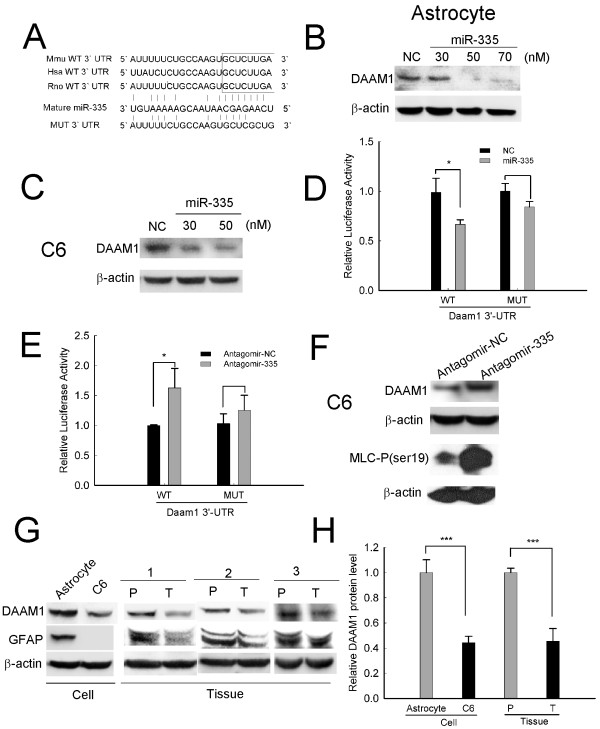
**Daam1 is a direct target of miR-335**. (A) Schematic representation of 3'-UTR of Daam1 mRNA with the putative miR-335 binding sequence. Mutation was introduced to the Daam1 3'-UTR sequence in the complementary site for the seed region of miR-335. (B, C) Effects of miR-335 on expression of endogenous DAAM1 protein. Western blot analysis was used to monitor DAAM1 protein expression in rat astrocytes (B) and C6 cells (C) 72 h after transfection with miR-335 mimics or negative control mimics (NC) at the indicated concentrations. (D, E) Analysis of luciferase activity. C6 cells were cotransfected with psiCHECK-2-wild type Daam1 3'-UTR/-mutant Daam1 3'-UTR (indicated as WT or MUT on the × axis), and (D) miR-335 mimics or (E) antagomir-335, luciferase activity was assayed 72 h after transfection. (F) Effects of antagomir-335 on expression of DAAM1 and phosphorylation of MLC (myosin light chain). C6 cells were transfected with 100 nM antagomir-335 or antagomir-NC, and applied for western blot analysis 72 h later. (G) DAAM1 protein is significantly downregulated in astrocytoma. Endogenous DAAM1 expression in C6 cells and human astrocytoma tissues were detected by western blot. GFAP expression was used as a biomarker of normal astrocytes. (H) Quantified protein levels of DAAM1 are shown as normalized by β-actin. Data represent the means ± SD of three independent experiments. Statistical differences compared with the controls are given as *, P < 0.05; **, P < 0.01; ***, P < 0.001.

To identify specific gene targets of miR-335 by which it might promote oncogenic behaviors, we searched diverse target prediction databases (TargetScan, Pictar) for theoretical target genes whose downregulation could mediate the observed effects of miR-335. Among all the putative targets, Daam1, Rasa1 and Map2 which we chose for our further study were known to play important roles in cell motility or proliferation[24,25]. The predicted miR-335 binding sites were shown (Figure 4A, Additional file 2 Figure S2). Interestingly, after introducing miR-335 into C6 cells and normal astrocytes, a dramatic downregulation of DAAM1 protein in a dose-dependent manner was concurrent with an unaltered Daam1 mRNA (Figure 4B, C, Additional file 2 Figure S3), indicating a posttranscriptional modulation of miR-335 on Daam1. In parallel experiments, no obvious changes of RASA1, and MAP2 protein levels could be detected (Additional file 2 Figure S4). Then, a consistent reduction of luciferase activity of the reporter that contained wild-type 3'-UTR of Daam1 mRNA was observed in the condition of extra miR-335 (Figure 4D), while the luciferase activity of mutant reporter was almost unaffected. In contrast, the addition of antagomir-335 in C6 cells increased luciferase activity (Figure 4E), associated with the upregulation of DAAM1 protein (Figure 4F up-panel). Importantly, the phosphorylation of MLC (myosin light chain) which is a downstream factor of DAAM1 and the direct trigger of apoptosis was strongly induced after antagomir-335 transfection (Figure 4F down-panel).

To further discern the regulatory role of endogenous miR-335 in DAAM1 protein expression, we analyzed the protein level of DAAM1 in C6 cells and normal astrocytes in which miR-335 level had been detected by qRT-PCR (Figure 1B). As expected, we observed a sharp reduction of DAAM1 protein in miR-335-overexpressed C6 cells (Figure 4G left-panel, 2H). Here, GFAP expression was used as a biomarker of primary cultured normal astrocytes. Importantly, this reciprocal expression pattern was further confirmed in a set of resected human astrocytoma tissue samples. As Figure 4G right-panel, 2H shown, DAAM1 was highly expressed in all of the respective adjacent paracancerous fields, but barely detectable in human astrocytoma tissues, although the degree of low-expression varied from one sample to another. These data indicate that miR-335 may inhibit the expression of Daam1 at posttranscriptional level by directly targeting the 3'-UTR of Daam1 mRNA.

### Knockdown of Daam1 mimics the oncogenic effects of miR-335 in C6 cells

**Figure 5:**
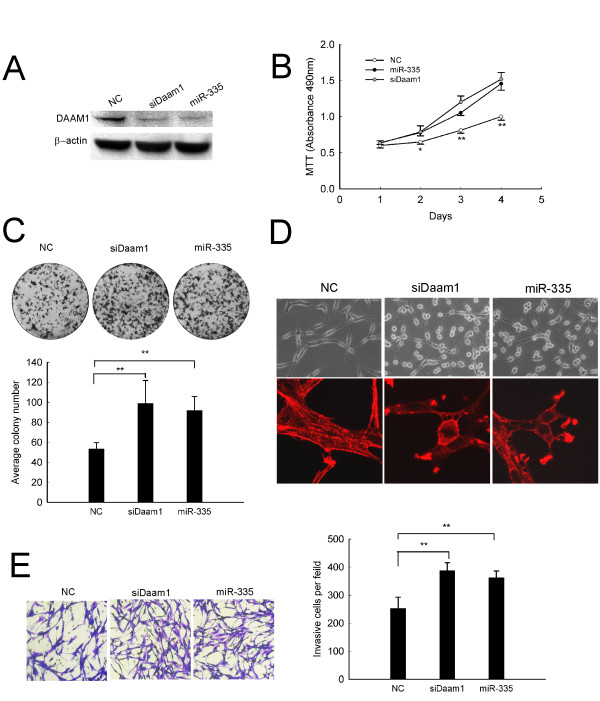
**SiDaam1 mimics the oncogenic effects of miR-335 in C6 cells**. (A) SiDaam1 efficiently inhibited the expression of DAAM1. Western blot was used to detect the expression of DAAM1 72 h after transfection with 50 nM siDaam1 or miR-335 mimics in C6 cells. (B) Cell viability was detected by MTT assay. (C) Effect of siDaam1 or miR-335 transfection on colony formation. (D) Morphologic alteration (up-panel) and phallotoxins stained actin rearrangement (down-panel). Cells transfected with siDaam1 or miR-335 adopted a stellate morphology and collapsed their actin stress fibers together with increased membrane ruffling. (E) Cell invasiveness was detected by transwell invasion assay. C6 cells were transfected with 50 nM siDaam1 or miR-335 mimics for the indicated times. Results represent the means ± SD for three repeats. (*, p < 0.05;**, p < 0.01). Original magnification in (D up-panel; E), 200 ×; (D down-panel), 630 ×.

To determine whether Daam1 is a functional target of miR-335 in malignant astrocytoma cells, we investigated whether reduction of Daam1 expression could mimic the growth and invasion-promoting effects of miR-335. As Figure 5A shown, knockdown of Daam1 (siDaam1) in C6 cells for 48 h resulted in a major reduction in DAAM1 protein, the repression effect was also observed by miR-335 transfection. Effective Daam1 silencing could dramatically stimulate viability and colony formation of C6 cells, which were similar to that of miR-335 transfection (Figure 5B, C). Moreover, both miR-335 and siDaam1 transfected cells showed a dramatic cytoskeleton rearrangement characterized by a loss of actin stress fibers and induction of actin-positive membrane ruffles (Figure 5D). Accordingly, an enhanced invasiveness was further observed both in miR-335 and siDaam1 treated cells (Figure 5E). These findings suggest that Daam1 repression may contribute to the miR-335-induced oncogenic features.

### Knockdown of Daam1 reverses the anti-tumor effects of antagomir-335 in C6 cells

**Figure 6:**
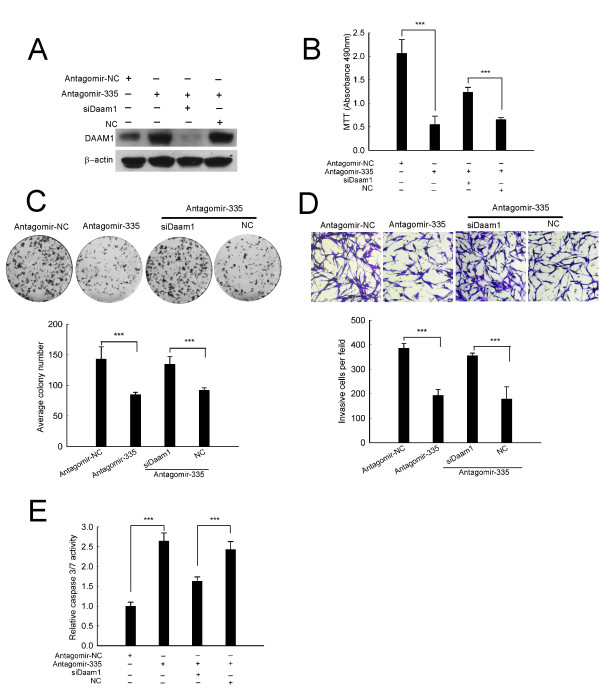
**SiDaam1 reverses the anti-tumor effects of antagomir-335 in C6 cells**. (A) SiDaam1 obviously abrogated upregulation of DAAM1 induced by antagomir-335 transfection. (B-E) siDaam1 counteracted the anti-tumor effects of antagomir-335. SiDaam1 significantly abrogated antagomir-335-induced growth arrest (B), repression of colony formation(C), invasion inhibition (D) and caspase3/7 activity (E). C6 cells were transfected with 50 nM siDaam1 and/or 100 nM antagomir-335 for the indicated times. Results represent the means ± SD for three repeats. (***, p < 0.001). Original magnification in (D), 200 ×.

We further examined whether Daam1 abrogation counteracts the anti-tumor effects of antagomir-335. As Figure 6A shown, siDaam1 effectively abolished the upregulation of DAAM1 induced by antagomir-335. Interestingly, the resulting Daam1 knockdown obviously rescued the antagomir-335-induced growth arrest, repression of colony formation and invasion (Figure 6B, C, D). Furthermore, siDaam1 transfectants also significantly decreased the caspase3/7 activity induced by antagomir-335(Figure 6E). Taken together, all these data suggest that Daam1 is potentially involved in miR-335-regulated growth and invasion.

### MiR-335 inhibition suppresses tumor growth *in vivo *

**Figure 7:**
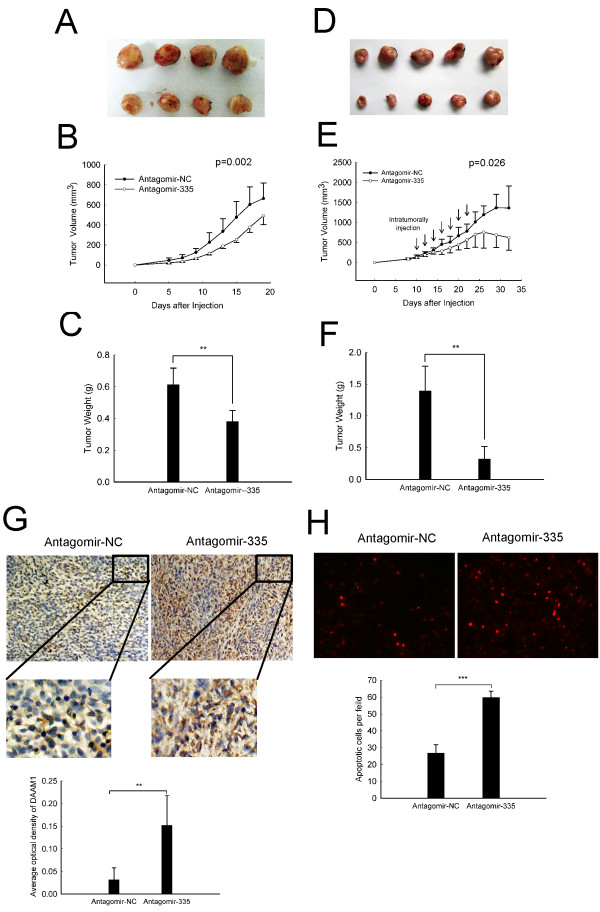
**MiR-335 inhibition suppresses tumor growth *in vivo***. (A-C) Pretreated effect of antagomir-335 on tumor formation in nude mouse xenograft model. (A)Antagomir-NC-transfected and antagomir-335-transfected C6 cells were s.c. injected into the left (up-panel) and right (down-panel) flanks of nude mice, respectively (n = 8). (B) Tumor volume was monitored during the time course of 19 days. (C) Tumor weight was detected at the end of the study. (D-H) Therapeutic effect of antagomir-335 on tumor growth in nude mouse xenograft model. (D) Tumors generated by C6 cells injection in nude mice were intratumorally injected with antagomir-NC (up-panel) and antagomir-335 (down-panel) for 2 weeks, respectively(n = 5). (E) Tumor volume was monitored during the time course of 4 weeks. (F) Tumor weight was detected at the end of the study. (G) DAAM1 expression was detected by immunohistochemistry. (H) Apoptotic cells were detected by TUNEL assay. Data represent the means ± SD. Statistical differences compared with the controls are given as **, P < 0.01; ***, P < 0.001. Original magnification in (G up-panel), 320 ×; Original magnification in (H), 400 ×.

Based on the *in vitro *studies, we hypothesized that abolition of miR-335 expression might have anti-tumor effects *in vivo*. To address this critical question, we used two *in vivo *xenograft models. In pretreated model, as shown in Figure 7A-C, pre-transfection of antagomir-335 into C6 cells led to a significant reduction in both tumor volume (Figure 7A, B) and weight (Figure 7C) compared with antagomir-NC transfectant. Furthermore, to determine whether inhibition of miR-335 may have therapeutic effects, C6 cells were injected into nude mice, and tumors thereby generated were treated with antagomir-335 or antagomir-NC for two weeks(Figure 7D). In line with the pretreated model, intratumorally injection of antagomir-335 dramatically inhibited tumor growth and weight (Figure 7E, F). Intriguingly, the ratio of the anterior flank metastasis was as high as 40% in antagomir-NC group; however, no metastasis was observed in antagomir-335 group (Additional file 2 Figure S5). Consistently, immunohistochemistry showed a marked increase of DAAM1 protein level in antagomir-335 treated group (Figure 7G). In addition, TUNEL assay showed an increased apoptosis in tumors administrated with antagomir-335, as compared to the control group (Figure 7H). All these results suggest that miR-335 may confer a survival advantage to astrocytoma cells and that it might be a novel potential therapeutic target for malignant astrocytoma therapy.

### Both miR-335 and siDAAM1 promote growth and invasion of human U87-MG astrocytoma cells

**Figure 8:**
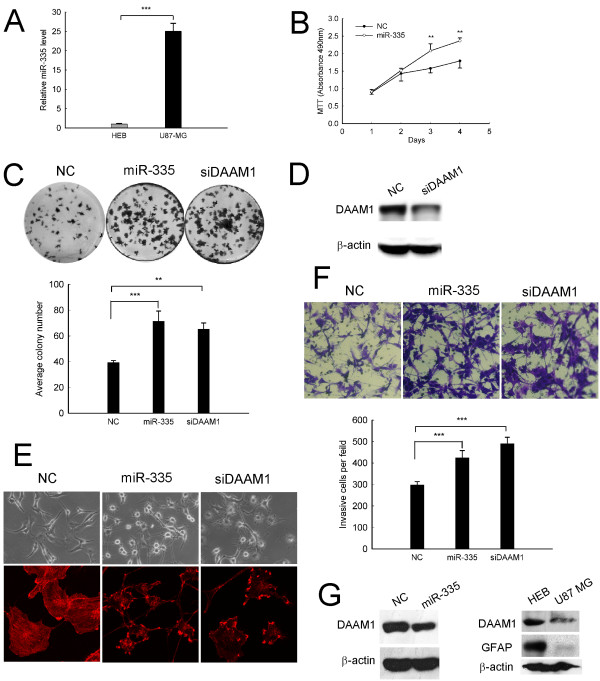
**Both siDAAM1 and miR-335 promote growth and invasion of human astrocytoma U87-MG cells**. (A) miR-335 expression in U87-MG and HEB cells was detected by real-time qRT-PCR. (B) Cell viability was detected by MTT assay. (C) Effect of siDAAM1 or miR-335 transfection on colony formation. (D) Effect of siDAAM1 transfection on DAAM1 protein expression. U87-MG cells were transfected with 50 nM siDAAM1 for 72 h. (E) Morphologic alteration (up-panel) and phallotoxins stained actin rearrangement (down-panel). Cells treated with siDAAM1 or miR-335 adopted a stellate morphology and collapsed their actin stress fibers together with increased membrane ruffling. (F) Cell invasiveness was detected by transwell invasion assay. (G left-panel) Western blot analysis was used to detect DAAM1 protein expression in U87-MG cells 72 h after transfection with 100 nM miR-335 mimics. (G right-panel) Endogenous DAAM1 expression in U87-MG and HEB cells was detected by western blot. GFAP expression was used as a biomarker of normal astrocytes. Results represent the means ± SD for three repeats. (*, p < 0.05;**, p < 0.01;***, P < 0.001). Original magnification in (E up-panel; F), 200 ×; (E down-panel), 400 ×.

To test whether our findings extend to human astrocytoma cells, we first compared miR-335 expression between human astrocytoma U87-MG cells and human normal astrocyte HEB cells. Consistent with the C6 cells, the expression of miR-335 was markedly upregulated in U87-MG astrocytoma cells (Figure 8A). Furthermore, ectogenic expression of miR-335 significantly promoted viability of U87-MG cells in a time-dependent manner (Figure 8B). And an enhanced colony formation was further observed (Figure 8C). Importantly, this colony-promoting effect was also detected by effective DAAM1 abrogation (Figure 8C, D). Moreover, cells transfected with miR-335 or siDAAM1 showed a loss of actin stress fibers, adopting more of a stellate morphology with an increase of actin-rich cell processes and membrane ruffles (Figure 8E). We also observed that both miR-335 and siDAAM1 efficiently accelerated the invasiveness of U87-MG cells *in vitro *(Figure 8F). In addition, the expression of DAAM1 was substantially downregulated after miR-335 transfection (Figure 8G left-panel). Moreover, the reciprocal expression pattern between endogenous miR-335 and DAAM1 was further confirmed in HEB and U87-MG cells (Figure 8G right-panel). All of the above findings together with those observed in C6 cells suggested that miR-335 might act as an oncomiRNA both in rat and human malignant astrocytoma pathgenesis.

### MiR-335 inhibition induces apoptosis and suppresses invasion in human U87-MG astrocytoma cells

**Figure 9:**
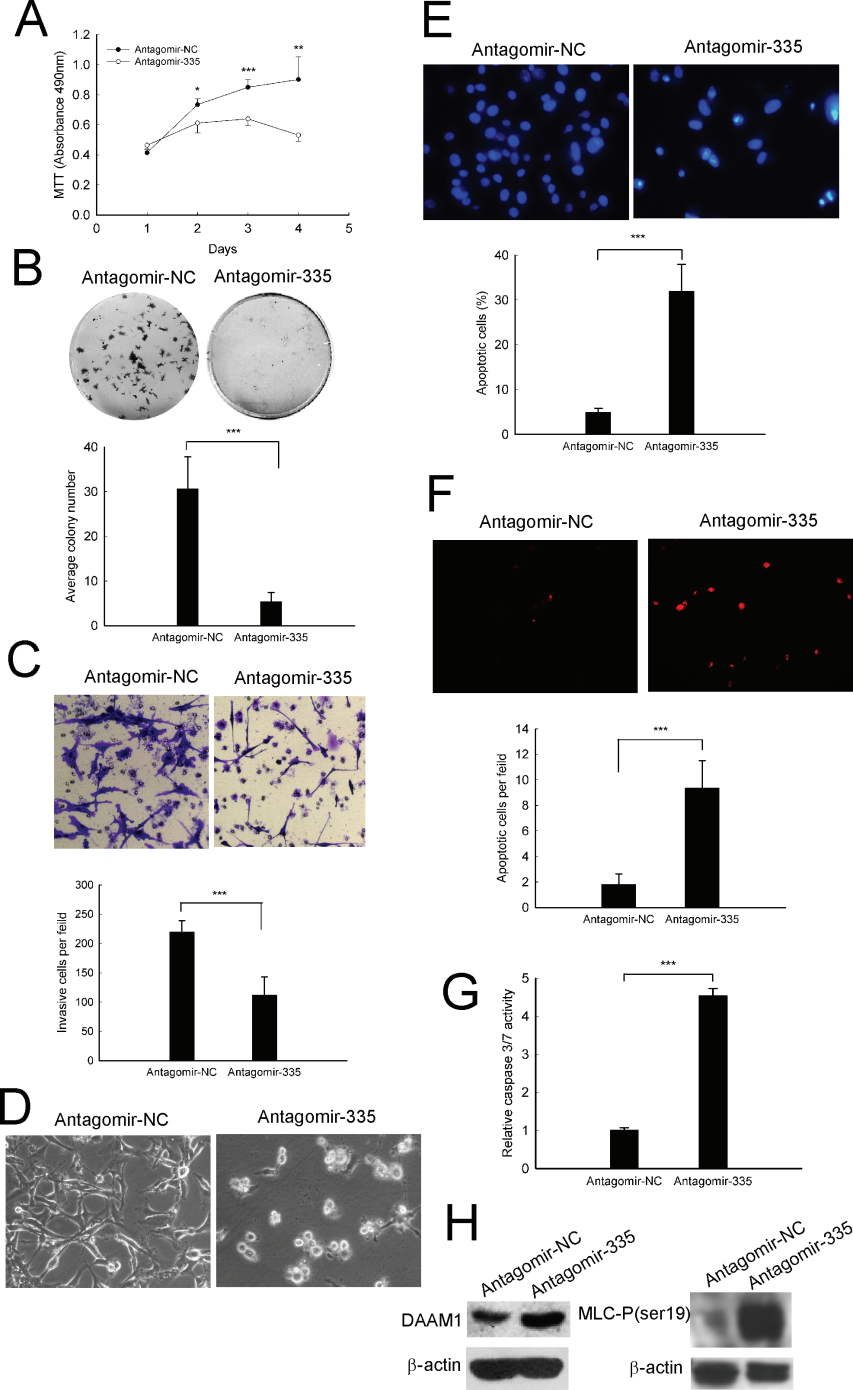
**MiR-335 inhibition induces apoptosis and suppresses invasion of human astrocytoma U87-MG cells**. (A) Time-dependent effect of antagomir-335 on cell viability. U87-MG cells were transfected with 100 nM antagomir-335 for the indicated times. (B) Effect of antagomir-335 transfection on colony formation. (C) Cell invasiveness was detected by transwell invasion assay. U87-MG cells were transfected with 100 nM antagomir-335 or antagomir-NC. Graph is the representative of three independent experiments. (D) Phase-contrast imaging and (E) Hoechest 33258 staining of U87-MG cells. (F) Apoptotic cells were detected by TUNEL assay. (G) Effect of antagomir-335 on caspase3/7 activity. U87-MG cells were transfected with 100 nM antagomir-335 or antagomir-NC for 72 h. (H) Effect of antagomir-335 on expression of DAAM1 (left-panel) and phosphorylation of MLC (right-panel) in U87-MG cells. Cells were transfected with 100 nM antagomir-335 or antagomir-NC for 48 h. Data represent the means ± SD. Statistical differences compared with the controls are given as *, P < 0.05; **, p < 0.01; ***, P < 0.001. Original magnification in (C, D, F), 200 ×; Original magnification in (E), 400 ×.

The anti-tumor effects of miR-335 abrogation were further investigated in U87-MG cells. As Figure 9A, B, C, shown, antagomir-335 strongly inhibited viability, colony formation and invasion of U87-MG cells. Moreover, after exposure to antagomir-335, the U87-MG cells displayed all of the apoptotic morphological alterations such as shrinkage of the cell, condensation of chromatin, and disintegration of the cell into small fragments (Figure 9D, E). Consistently, TUNEL assay showed an increased apoptosis in antagomir-335 transfected U87-MG cells (Figure 9F). We also observed caspase 3/7 activity was markedly enhanced after 72 h antagomir-335 tranfection, confirming miR-335 abrogation could induce caspase-mediated apoptosis in human astrocytoma cells (Figure 9G). In addition, the expression of DAAM1 was substantially upregulated after antagomir-335 transfection (Figure 9H left-panel). Moreover, the phosporylation of MLC (myosin light chain) was also highly induced (Figure 9H right-panel). All of the above findings seem to demonstrate that antagomir-335 is able to induce apoptosis and suppress invasion in both rat C6 and human U87-MG astrocytoma cells, which indicates the anti-tumor effects of miR-335 abrogation are evolutionarily conserved from rat to human in malignant astrocytoma.

## Discussion

Malignant astrocytomas are one of the leading causes of cancer deaths in central nervous system characterized by unlimited proliferation and progressive local invasion [1,26]. Unfortunately, the underlying molecular mechanisms resulting in astrocytomagenesis and local invasion remain obscure so far and stand for the major obstruction in finding novel therapeutic strategies[27]. In this study, we found that miR-335 targeted a potential tumor suppressor Daam1, which promoted several oncogenic features such as growth and invasion in astrocytoma cells. Furthermore, miR-335 inhibition could effectively suppress growth and induce apoptosis of astrocytoma cells both *in vitro *and *in vivo*. Importantly, the anti-tumor effects of miR-335 abrogation also extended to human astrocytoma cells. Thus, miR-335 might act as an evolutionarily conserved oncomiRNA in astrocytoma pathogenesis and represent a potential therapeutic target for this highly aggressive and, as yet, therapy-refractory tumor.

Genetic aberration patterns specific for different grades of astrocytoma have been defined previously[12]. Gain of chromosome 7 with a hot spot at 7q32 appears to be the most prominent aberration and an early event in tumorigenesis of astrocytoma, whereas in glioblastoma multiforme, gain of 7p12 seems to be the most frequently affected band on chromosome 7[12]. We found 8 expressed miRNAs (miR-593, miR-129-1, miR-335, miR-182, miR-96, miR-183, miR-29a, miR-29b-1) reside on 7q32, some of which have been investigated, either as oncogenes or tumor suppressor genes [13-15]. It is becoming increasingly clear that chromosomal abnormalities and/or epigenetic events contribute to miRNA deregulation[28]. Our data showed that the intronic miR-335, flanked by *MEST *imprinting gene in the 7q32.2 region, was highly expressed in astrocytoma cell lines and tissues. Intriguingly, genomic copy number analysis revealed statistically significant amplification of miR-335 locus in U87-MG cell line and II-III grade malignant astrocytoma tissues (Additional file 2 Figure S6). Moreover, we also determined the miR-335 expression level in glioblastoma multiforme T98G cell line, and found that it was slightly upregulated compared to HEB (Additional file 2 Figure S7). Loss of function study also showed that inhibition of miR-335 in T98G cells had little effect on their growth (Additional file 2 Figure S8). Therefore, we hypothesize that the observed increased expression of miR-335 may partially result from 7q32.2 regional amplification, and miR-335 may act as an early promoter and a biomarker during tumorigenesis of astrocytoma.

To date, a panel of miRNA mutations or deletions have been reported, either tumor suppressors or oncogenes in multiple human cancers[5], including malignant astrocytomas[29]. Even more, impairment of microRNA regulatory network is considered as one of the key mechanisms in astrocytoma pathogenesis[30].With regard to miR-335, it is normally expressed in a variety of human tissues and deregulated in several types of tumors[31-34], suggesting complex biological roles of this miRNA during tumorigenesis. Of particular interest, miR-335 was recently reported to suppress metastasis of human breast cancer *via *targeting of SO × 4 and tenascin C, however, without affecting its proliferation [17]. This finding is apparently in contradiction with the function of miR-335 as an invasion promoter in malignant astrocytomas. Due to the fact that one miRNA can regulate more than one target gene, it is possible to speculate the selection of genes that would make major contributions to the phenotypes induced by the miRNA may depend on the cellular microenvironment. Therefore, we propose that the downregulation of DAAM1 induced by miR-335 may play a predominant role in mediating the pro-invasion effect of miR-335 in astrocytoma cells. Furthermore, it is also demonstrated that miR-335 regulates Rb1 and controls cell proliferation in a p53-dependent manner[18]. MiR-335 drives hyperproliferation in the absence of p53 and induces apoptosis and/or cell cycle arrest in the presence of wide-type p53[18]. In our study, we showed that miR-335 strongly promoted growth of C6 and U87-MG astrocytoma cells. However, it is well established that both C6 and U87-MG cells express wild type (wt)-p53 [35,36]. Moreover, we further detected the effect of miR-335 on Rb1 expression in astrocytoma cells. Surprisingly, transient transfection of C6 and U87-MG cells with miR-335 efficiently increased the Rb1 protein levels, as detected by Western blotting (Additional file 2 Figure S9). This discrepancy is likely due to the difference in cell context, and suggests that altered expression of this miRNA may have diverse effects in tumor cells. In fact, the interaction between miRNA and its target mRNA can be controlled by many factors. For example, recent studies have shown that there are AU-rich elements (AREs) resided in the vicinity of the microRNA target sites (seed sequence), and both AREs and seed sequence are highly conserved throughout evolution in mRNA 3'UTRs [37]. Intriguingly, two RNA binding proteins HuR and Dnd1 have been identified to counteract the function of miRNAs by binding AREs and blocking miRNAs from associating with their target sites[38,39]. Even more, both miR-369-3 and let-7 have been shown to activate target gene expression on cell cycle arrest by recruiting specific proteins to AREs[37]. Thus, it is reasonable to assume that astrocytoma cell-specific factors bind and counteract miR-335 function in these cells. Further experiments to clarify this discrepancy would be of great interest.

Dishevelled-associated activator of morphogenesis 1 (Daam1), a member of the formin protein family acting downstream of WNT signaling, plays an important role in regulating the actin cytoskeleton *via *mediation of linear actin assembly [40]. Previous functional studies of Daam1 suggest its essential roles in promoting proper cell polarization, migration, proliferation and tissue morphogenesis during embryonic development[41]. Inhibition of Daam1 results in an increase in cell migration of parietal endoderm [42] while activation of Daam1 suppresses endothelial cell proliferation, migration and angiogenesis[43]. It is also demonstrated that stably overexpressing Daam1 enhances myosin IIB stress fiber network which opposes cell migration[41]. Additionally, Daam1 has been linked to the control of cell behaviors by regulating downstream Rho/ROCK [42]. It is well established that inhibition of ROCK can promote motility of astrocytoma cells *via *actin rearrangement[44]. In line with these observations, our data showed that both miR-335 and knockdown of Daam1 (siDaam1) efficiently promoted invasion of C6 and U87-MG astrosytoma cells, and an alteration of cell morphology as well as a loss of actin stress fibers together with induction of actin-positive membrane ruffles were also observed, indicating that the actin rearrangement may contribute to the pro-invasive effect of miR-335 in astrocytoma cells. Furthermore, ROCK is recognized as a major regulator of the morphological events that occur during the execution phase of apoptosis, including cell contraction, dynamic membrane blebbing, nuclear disintegration, and fragmentation of apoptotic cells into apoptotic bodies. It is reported that activation or overexpression of ROCK increases MLC (myosin light chain) phosphorylation and subsequently results in actomyosin contractility, membrane blebbing and apoptosis[45,46]. In our study, we found that antagomir-335 dramatically upregulated DAAM1 protein, meanwhile, the phosphorylation of MLC was also extremely stimulated both in C6 and U87-MG cells. These results suggest that antagomir-335 induced apoptosis may partially through the increase of DAAM1 which, in turn enhances MLC phosphorylation. In addition, it is noteworthy that siDaam1 was able to mimic the oncogenic behaviors of miR-335; however, it could only counteract partially the anti-tumor effects of antagomir-335. Therefore, it is likely that miR-335 has effects independent of DAAM1. Alternatively, it would be reasonable that the downregulation of DAAM1 could simply cooperate with the concomitant attenuation of other miR-335 targets.

Besides *in vitro *study, we also found that antagomir-335 could effectively inhibit tumor growth in both pretreated and therapeutic xenograft models. Similar to targeted therapies that tackle a gain-of-function in cancer, such as EGFR inhibitors, the introduction of the antagonist specific to oncomiRNA miR-335 interferes with the oncogenic properties of astrocytoma cells and induces therapeutic responses. This "miRNA targeting therapy" has been given more and more attention and successfully demonstrated in several human diseases[47-50], indicating a potential application of this approach in clinical settings. While our results presented here suggest that antagomir-335 may prove useful in the treatment of astrocytoma, the challenge for this approach will be the successful delivery of the miRNA antagonist to the tumor cells. Local delivery of synthetic antagomir-335 by intratumoral injections resulted in strong inhibition of tumor growth. Nevertheless, this delivery route might be inadequate in a clinical setting as peripheral tumor cells remained present. Therefore, a delivery technology that facilitates universal access to all tumor cells, such as a systemic delivery route, might be needed to make the therapy more efficacious.

## Conclusions

In summary, our present study uncovered an oncogenic function of miR-335 resided on chromosome 7q32.2 that amplified frequently in astrocytoma, which may provide a novel insight to its molecular etiology. This effect may be caused by WNT/PCP signaling inhibition ascribed to the restrain of DAAM1 protein. Of note, miR-335 abrogation displayed notable anti-tumor effects both *in vitro *and *in vivo*. Significantly, the anti-tumor effects also extended to human malignant astrocytoma, indicating the evolutionarily conserved function of miR-335 and a potential application of targeting miR-335 in the therapy of malignant astrocytoma.

## Abbreviations

miRNA: microRNA; oncomiR: oncogenic miRNA; DAAM1: disheveled-associated activator of morphogenesis 1; siRNA: small interfering RNA; UTR: untranslated region; FBS: fetal bovine serum; GFAP: glial fibrillary acid protein; MTT: 3-(4,5-dimethylthiazol-2-yl)-2,5-diphenyl-tetrazolium bromide; SC: subcutaneous; ROCK: Rho-associated kinase; MLC: myosin light chain; MEST: mesoderm-specific transcript.

## Competing interests

The authors declare that they have no competing interests.

## Authors' contributions

MS, SW, and XZ contributed equally to this work; MS and GY designed research; MS, SW, XZ, YZ, YL, TL and DX performed research; MS, WZ and GY analyzed data; and MS, HL, WZ, YO, XL, and GY wrote the paper. All authors read and approved the final manuscript.

## Supplementary Material

Additional file 1 **Table S1**. Patients' characteristics of the tumor samples used in our experiment. click here for file

Additional file 2 **Supplementary Figures.** Figure S1. MiR-335 fails to transform but enhances viability and invasiveness of normal astrocytes. (A) Cell viability was detected by MTT assay. (B) Cell invasiveness was determined by transwell assay. (C) Effect of miR-335 transfection on colony formation. Cells were transfected with 50 nM miR-335 mimics for the indicated times. Results represent the means ± SD for three repeats. (*, *P *< 0.05;**, *P *< 0.01). Original magnification in (B), 200 ×. Figure S2. Putative miR-335 binding sites in the 3'-UTR of respective genes. Figure S3. Effect of miR-335 overexpression on endogenous Daam1 mRNA level. Rat normal astrocytes were transfected with 50 nM miR-335 mimics for 48 h. Daam1 mRNA was detected by qRT-PCR. Figure S4. Effect of miR-335 overexpression on endogenous RASA1 and MAP2 protein levels. C6 cells were transfected with 50 nM miR-335 mimics for 72 h. Western blot was used to detect the protein levels of respective genes. Figure S5. Effect of antagomir-335 on tumor metastasis in nude mouse xenograft model. The same side anterior flank metastasis was indicated by arrows (up-panel) in antagomir-NC group. The ratio of metastasis was quantified in two groups (down-panel). Figure S6. Genomic copy number analysis reveals statistically significant amplification of miR-335 locus in U87-MG cell line and II-III grade malignant astrocytoma tissues. Quantitative genomic real-time PCR was performed on DNA from HEB, U87-MG cell lines as well as normal brain (N) and II-III grade malignant astrocytoma tissues. Figure S7. MiR-335 expression analysis in human astrocytes HEB and glioblastoma multiform T98G cells. Figure S8. Effect of miR-335 abrogation on cell growth. T98G cells were transfected with 100 nM antagomir-335 for the indicated times. Cell viability was detected with MTT assay. Figure S9. Effect of miR-335 on Rb1 expression in C6 and U87-MG astrocytoma cells. Cells were transfected with 50 nM miR-335 for 48 h. Rb1 protein was detected by Western blot. click here for file
