# Supplementary figures and images for: Targeting oncogenic miR-335 inhibits growth and invasion of malignant astrocytoma cells
Source: Mol Cancer. 2011 May 19;10:59. doi: 10.1186/1476-4598-10-59 (PMC3129318; doi:10.1186/1476-4598-10-59)

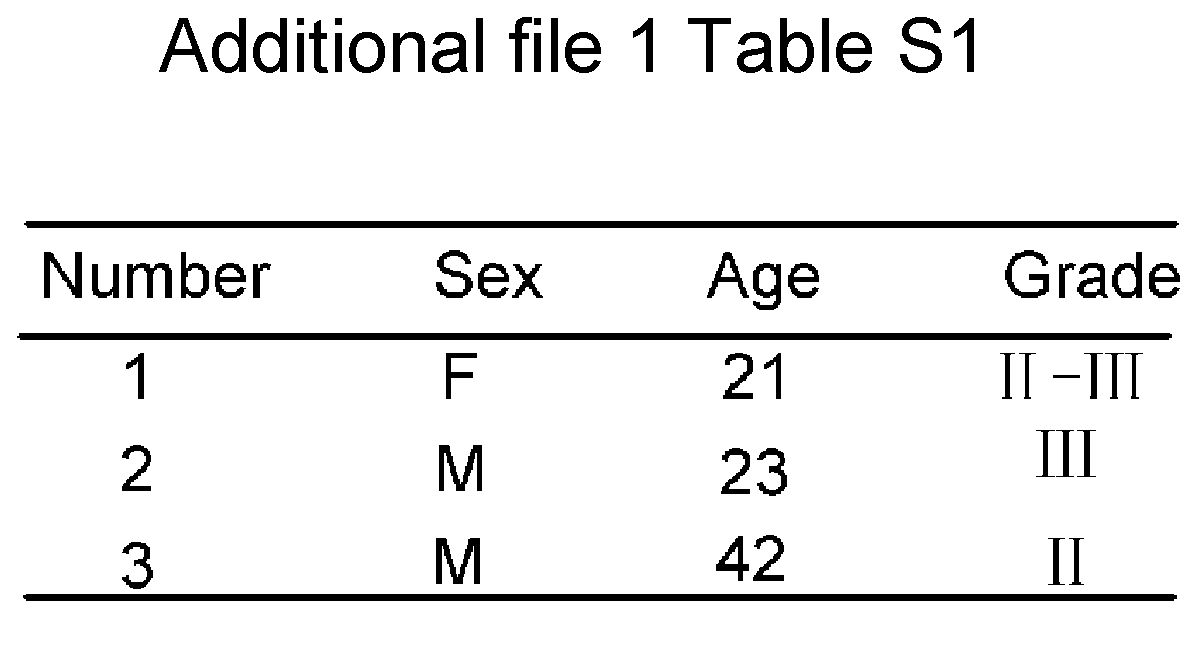

Supplement: Additional file 1 — Table S1. Patients' characteristics of the tumor samples used in our experiment. [file 1476-4598-10-59-S1.tiff]

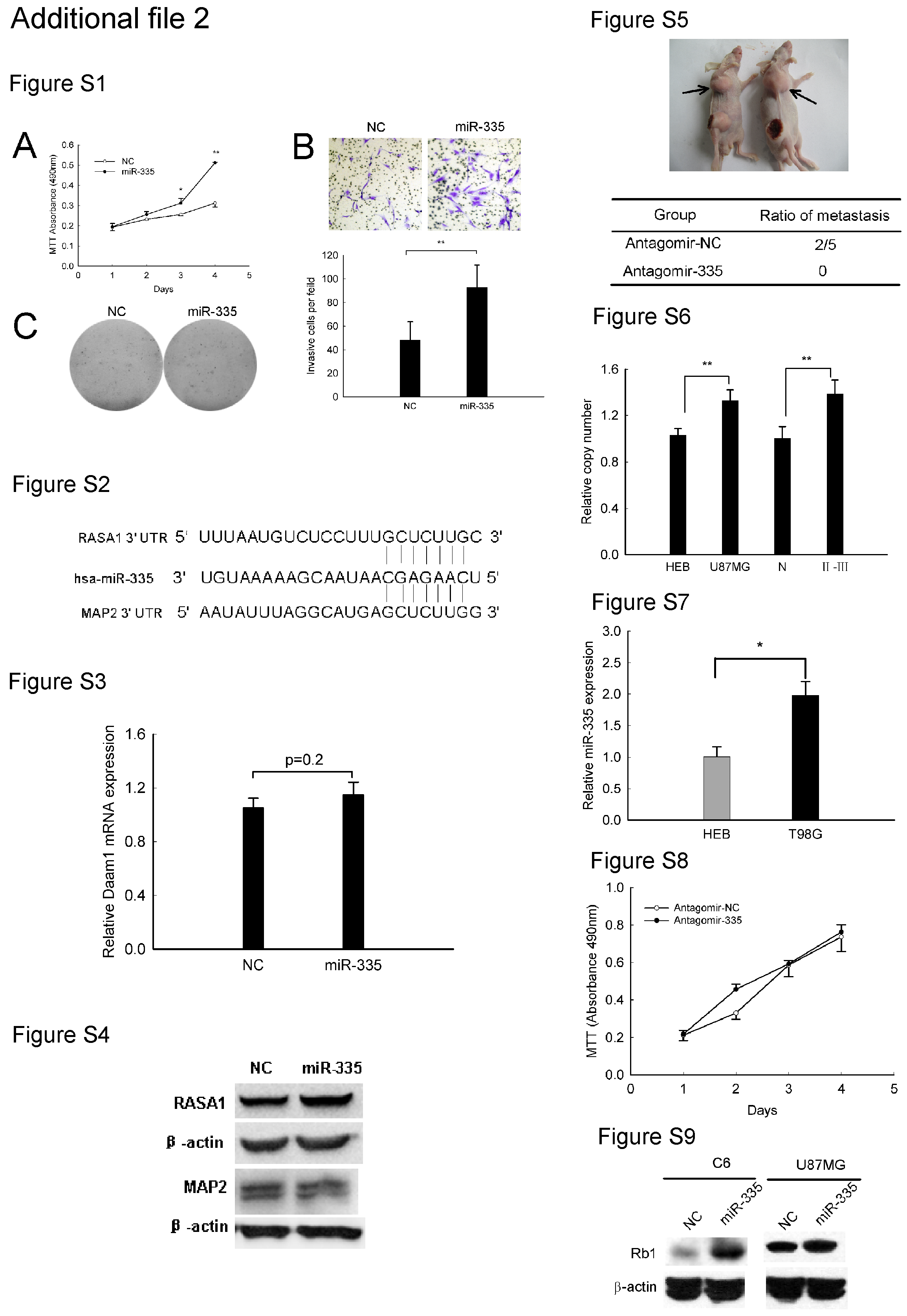

Supplement: Additional file 2 — Supplementary Figures. Figure S1. MiR-335 fails to transform but enhances viability and invasiveness of normal astrocytes. (A) Cell viability was detected by MTT assay. (B) Cell invasiveness was determined by transwell assay. (C) Effect of miR-335 transfection on colony formation. Cells were transfected with 50 nM miR-335 mimics for the indicated times. Results represent the means ± SD for three repeats. (*, P < 0.05;**, P < 0.01). Original magnification in (B), 200 ×. Figure S2. Putative miR-335 binding sites in the 3'-UTR of respective genes. Figure S3. Effect of miR-335 overexpression on endogenous Daam1 mRNA level. Rat normal astrocytes were transfected with 50 nM miR-335 mimics for 48 h. Daam1 mRNA was detected by qRT-PCR. Figure S4. Effect of miR-335 overexpression on endogenous RASA1 and MAP2 protein levels. C6 cells were transfected with 50 nM miR-335 mimics for 72 h. Western blot was used to detect the protein levels of respective genes. Figure S5. Effect of antagomir-335 on tumor metastasis in nude mouse xenograft model. The same side anterior flank metastasis was indicated by arrows (up-panel) in antagomir-NC group. The ratio of metastasis was quantified in two groups (down-panel). Figure S6. Genomic copy number analysis reveals statistically significant amplification of miR-335 locus in U87-MG cell line and II-III grade malignant astrocytoma tissues. Quantitative genomic real-time PCR was performed on DNA from HEB, U87-MG cell lines as well as normal brain (N) and II-III grade malignant astrocytoma tissues. Figure S7. MiR-335 expression analysis in human astrocytes HEB and glioblastoma multiform T98G cells. Figure S8. Effect of miR-335 abrogation on cell growth. T98G cells were transfected with 100 nM antagomir-335 for the indicated times. Cell viability was detected with MTT assay. Figure S9. Effect of miR-335 on Rb1 expression in C6 and U87-MG astrocytoma cells. Cells were transfected with 50 nM miR-335 for 48 h. Rb1 protein was detected by Western blo [file 1476-4598-10-59-S2.tiff]
